# Speech Misperception: Speaking and Seeing Interfere Differently with Hearing

**DOI:** 10.1371/journal.pone.0068619

**Published:** 2013-07-03

**Authors:** Takemi Mochida, Toshitaka Kimura, Sadao Hiroya, Norimichi Kitagawa, Hiroaki Gomi, Tadahisa Kondo

**Affiliations:** NTT Communication Science Laboratories, Nippon Telegraph and Telephone Corporation, Atsugi, Japan; UNLV, United States of America

## Abstract

Speech perception is thought to be linked to speech motor production. This linkage is considered to mediate multimodal aspects of speech perception, such as audio-visual and audio-tactile integration. However, direct coupling between articulatory movement and auditory perception has been little studied. The present study reveals a clear dissociation between the effects of a listener’s own speech action and the effects of viewing another’s speech movements on the perception of auditory phonemes. We assessed the intelligibility of the syllables [pa], [ta], and [ka] when listeners silently and simultaneously articulated syllables that were congruent/incongruent with the syllables they heard. The intelligibility was compared with a condition where the listeners simultaneously watched another’s mouth producing congruent/incongruent syllables, but did not articulate. The intelligibility of [ta] and [ka] were degraded by *articulating* [ka] and [ta] respectively, which are associated with the same primary articulator (tongue) as the heard syllables. But they were not affected by *articulating* [pa], which is associated with a different primary articulator (lips) from the heard syllables. In contrast, the intelligibility of [ta] and [ka] was degraded by *watching* the production of [pa]. These results indicate that the articulatory-induced distortion of speech perception occurs in an articulator-specific manner while visually induced distortion does not. The articulator-specific nature of the auditory-motor interaction in speech perception suggests that speech motor processing directly contributes to our ability to hear speech.

## Introduction

Speech perception is a multisensory process. Seeing mouth movement affects a listener’s auditory speech perception. Auditory [ba] combined with visual [ga] is typically heard as [da], with other audio-visual combinations generating perceptual confusion (i.e., the McGurk effect [1,2]). Tactile stimulation also variously affects speech perception [3–7]. How is information from different sensory modalities integrated into a unitary speech percept? The motor representation may underlie the auditory, visual and tactile speech information, and mediate their integration.

Theoretically, speech perception has long been thought to be linked to speech motor control [8–10]. Recent neurophysiological and neuroimaging studies have reported that auditory speech perception activates motor-related neural circuits, which are invoked to produce the same speech [11–13]. These findings suggest that the somatotopic motor representation of speech organs such as the lips and tongue is involved in speech perception [14]. Another study has revealed that seeing speech-related lip movements as well as hearing speech modulates the cortical excitability of the lip motor area [15]. Thus an increasing amount of evidence has shown that perception of others’ speech activates the corresponding motor circuits in the listener’s or observer’s brain.

However, it still remains unclear whether the involvement of motor-related areas is critical for speech perception. A group of patients with frontal brain damage and non-fluent speech, patients undergoing left-hemispheric anesthesia resulting in speech arrest [16], and even patients with bilateral anterior opercular cortex lesions causing anarthria [17], show intact speech perception performance. The motor account of speech perception can also be questioned considering evidence of categorical speech discrimination by pre-lingual infants [18] and by animals [19] (see 20–22 for reviews).

One way of studying the essentiality of the motor process in speech perception is to examine the reverse contribution of speech motor control to listening ability. Sams et al. have demonstrated that the perception of auditory syllables ([pa] and [ka]) can be disturbed or improved, respectively, when the listener silently articulates incongruent or congruent syllables as well as when he/she watches others’ mouths producing those syllables [23]. They concluded that the visual and articulatory disturbance effects on hearing discordant syllables share a common underlying mechanism. However, they only tested a single discordant pair (visual/articulatory [ka] with auditory [pa]) before drawing their conclusion.

In the present study, to clarify whether the listener’s own articulatory movement and another’s visual speech affect speech perception in the same manner, we examined how the syllable intelligibility of [pa], [ta], and [ka] changes with silent articulation and with visual mouth movement of congruent/incongruent syllables. The phonemes [p], [t], and [k] are all produced by the complete closure of the oral passage and its sudden release, but the speech organs mediating the productions are different [24]. The lips control the closure and release when articulating [p], whereas the tongue moves when articulating [t] and [k]. A similar contrast exists in the visual domain, that is, visual [p] looks very different from [t] and [k], whereas there is no such striking visual difference between [t] and [k]. Given that audio-visual interference should be sensitive to the visual divergence from the auditory information, the interaction between [p] and [t] and between [p] and [k] will be more prominent than between [t] and [k]. If the between-phoneme distance is common to the visual and articulatory domains, the audio-articulatory interaction might occur in the same manner as the audio-visual interaction, as Sams et al. have suggested [23]. However, our experimental results revealed interesting contrasts in syllable intelligibility when articulating and watching incongruent syllables.

## Materials and Methods

### Participants

Ten healthy adults (four males) aged 18 to 40 years (mean age +/- SD = 26.3 +/- 7.5 years) participated in the experiment. All the participants were native speakers of Japanese and exhibited no obvious speech difficulties as judged by the experimenters. They were naïve as to the purpose of the experiment.

### Ethics Statement

The study conformed to The Code of Ethics of the World Medical Association (Declaration of Helsinki) and was approved by the NTT Communication Science Laboratories Research Ethics Committee. All the participants gave their written informed consent to participating in the study.

### Tasks

**Figure 1:**
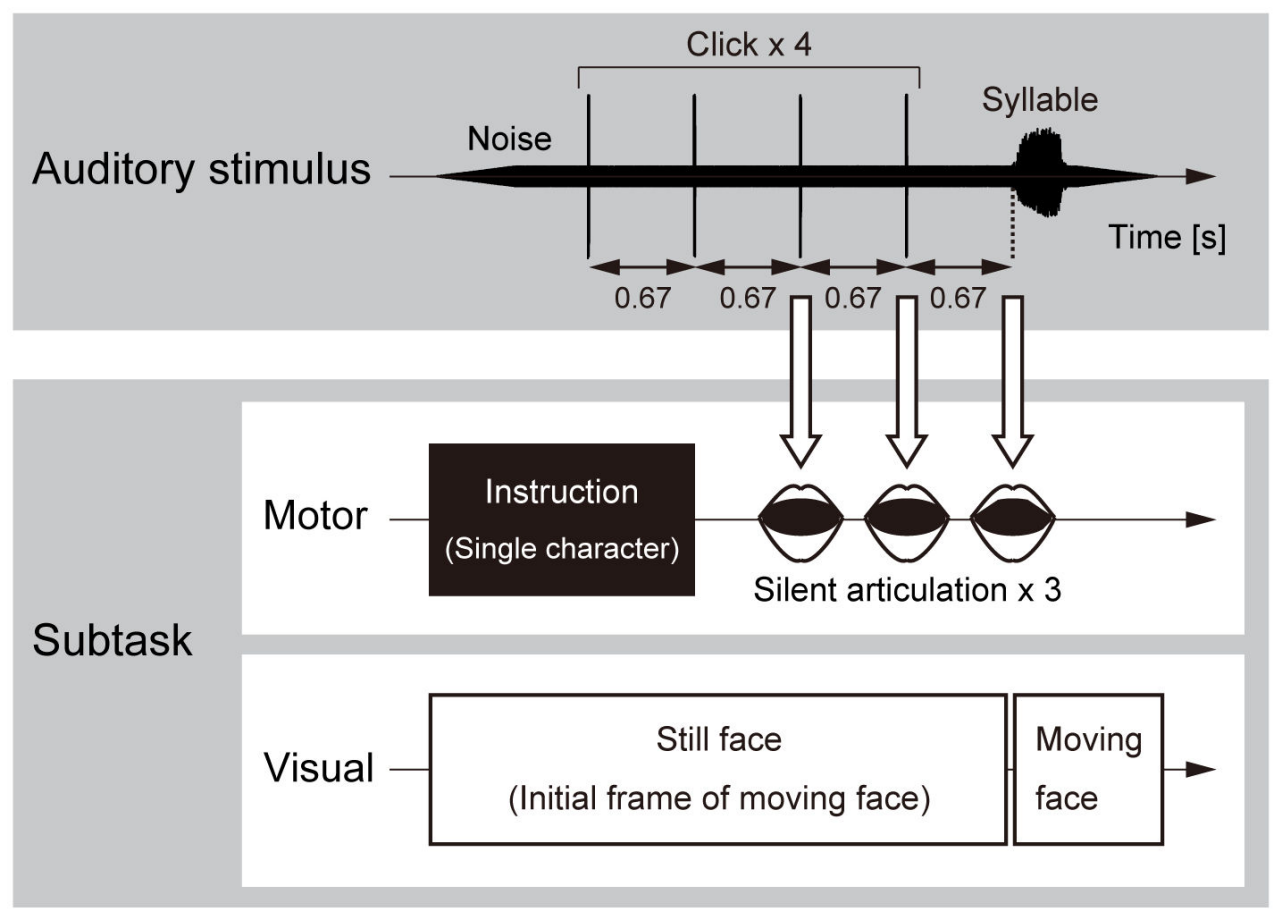
Auditory stimulus and subtask. The auditory stimulus was embedded in white noise to exclude the possibility of participants hearing their own speech under the motor condition, where the signal-to-noise ratio was set at 5 dB. The noise was faded in and out linearly over 0.5 s. The stimulus was preceded by four clicks at 0.67 s intervals, which provided the participants with a cue to silently articulate a syllable under the motor condition. Under the motor condition, the syllables to be articulated by the participants were first presented visually in Japanese characters, which then disappeared at the second click. The participants silently articulated syllables three times in time with the third and fourth clicks and the onset of the stimulus. Under the visual condition, videos of a speaker’s face producing syllables were presented, which were synchronized with the auditory stimulus. The initial frame of each video was presented from the noise onset to the stimulus onset.

Participants were asked to identify the syllables they heard under the following subtask conditions (Figure 1): silently articulating congruent/incongruent syllables (motor condition), watching videos of a speaker’s face producing congruent/incongruent syllables (visual condition), as well as without a subtask (control condition). In the motor condition, the participants were explicitly instructed to pronounce the syllables with as little audible sound as possible with a minimum amount of exhaling and without vibrating the vocal cords, while moving the lips and tongue as much as possible.

To ensure fair and impartial identification of the auditory target syllables ([pa], [ta], and [ka]), we added four other syllables ([ba], [da], [ga], and [a]) to the auditory stimuli and employed a seven-alternative forced-choice (7AFC) task, rather than using a 3AFC task. As regards the motor condition, silent articulations of [ba], [da], and [ga] were omitted from the subtask because voiced consonants are difficult to pronounce without vocal fold vibration. As regards the visual condition, videos of [ba], [da], and [ga] were omitted because they look similar to those of [pa], [ta], and [ka], respectively. A video of [a] was also omitted from the visual subtask because it did not include substantial movements of the lips and tongue.

### Stimuli

All the auditory stimuli were recordings produced by a male speaker, and digitized at 44100 Hz. The auditory syllables were presented to the participants via headphones (Sennheizer HD280Pro) at a level of 60 dBSPL with white noise in order to exclude the possibility of the participants hearing their own speech under the motor condition. The signal-to-noise ratio (SNR) was 5 dB. The beginning and end of the noise were faded in and out, respectively, over 0.5 s. The auditory syllables were preceded by four clicks (interclick interval of 0.67 s), which provided the participants with a cue to silently articulate a syllable under the motor condition.

In each trial under the motor condition, a Japanese character representing one of the four syllables, [pa], [ta], [ka], or [a], was visually presented on a front display at the onset of the white noise and removed at the second click. Participants were asked to silently articulate the indicated syllable three times while seeing a blank screen, in time with the third and fourth clicks and the onset of the auditory stimulus.

In each trial under the visual condition, a video of a speaker’s face pronouncing one of the three syllables, [pa], [ta], or [ka], was presented on an LCD monitor (EIZO FlexScan L66, 75Hz refresh rate) placed 55 cm in front of the participant’s eyes. The video was played at a frame rate of 30 fps with a frame size of 22 x 18 cm. The onset of the auditory stimulus was aligned with the onset of the syllable in the audio track associated with the video. (The audio track was not presented to the participants.) Prior to the video presentation, the initial frame of the video was presented at the onset of the white noise and it remained until the onset of the auditory stimulus.

### Experimental procedures

The experimental session for each participant consisted of two familiarization phase blocks followed by eleven test phase blocks. In the familiarization phase, the participants performed one control condition block and then one motor condition block. In the test phase, the participants performed five sets of one motor condition block and one visual condition block, with the order of the two blocks within each set randomized and counterbalanced for each participant. All the participants performed one control condition block at the end of the test phase. During the experimental session, the participants took short breaks between blocks. Each trial was initiated when the participants entered their response to the previous trial.

Each of the five blocks under the motor condition consisted of 84 trials in which each of the 28 different trials (7 auditory syllables x 4 subtask syllables) was performed three times. Each of the five blocks under the visual condition consisted of 63 trials in which each of the 21 different trials (7 auditory syllables x 3 subtask syllables) was performed three times. One block under the control condition consisted of 105 trials in which each of the seven auditory syllables was presented 15 times. The order of the trials in each block was randomized for each participant.

### Data analysis

For each auditory stimulus under each subtask condition, 15 responses were collected from which the correct response rate was calculated as a measure of syllable intelligibility. The correct response rates under the control (auditory only) conditions were then subtracted from their corresponding rates under the motor and visual conditions. These unbiased rates were compared using a three-way repeated-measures analysis of variance (ANOVA) with condition (motor/visual), subtask syllable (pa/ta/ka) and stimulus (pa/ta/ka) as within-subjects factors.

## Results

**Figure 2:**
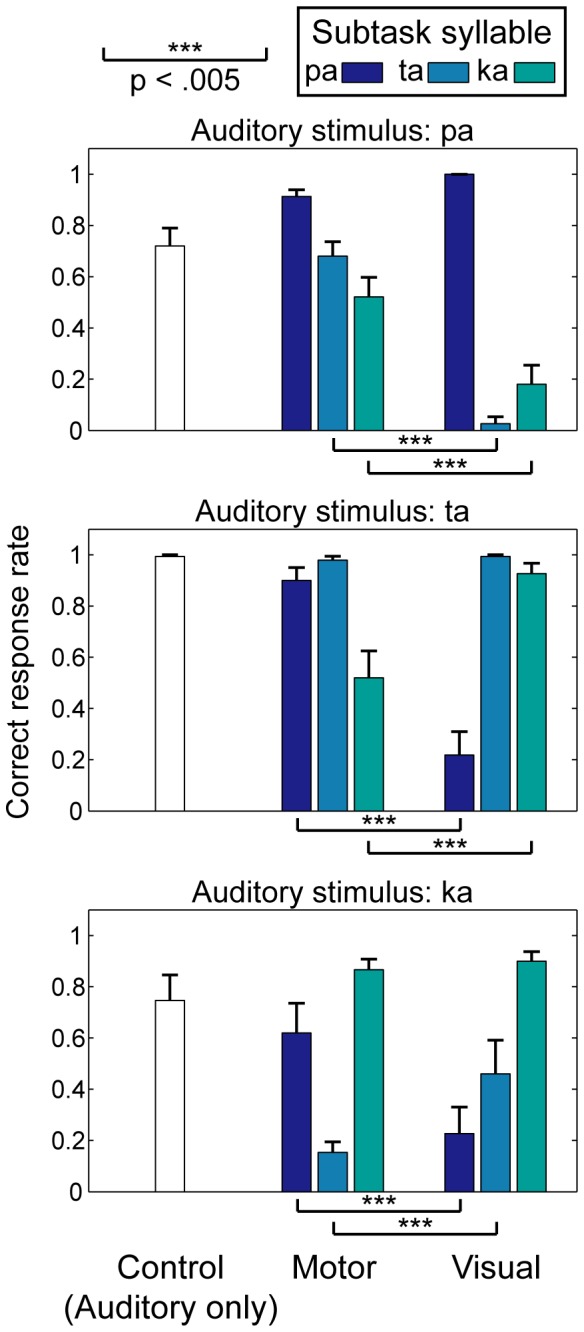
Syllable intelligibility. The mean and standard error (N = 10) of the correct response rates for auditory stimuli [pa], [ta], and [ka] (from top to bottom panels) are shown. Each color indicates a subtask syllable ([pa], [ta], and [k]) under motor (silently articulating) and visual (watching mouth) conditions. The open bars represent the control (auditory-only) condition.

The correct response rates for [pa], [ta], and [ka] under all conditions are shown in Figure 2. The imperfect perception under the control condition (0.72, 0.99, and 0.75 for [pa], [ta], and [ka], respectively) was due to the background noise presented in order to prevent participants from hearing their own speech under the motor condition. The higher control level for [ta] compared with [pa] and [ka] replicated the results found in an earlier study ( [25], +6 dB SNR condition). The correct response rates under the motor and visual conditions, from which the corresponding rates under the control (auditory only) condition were subtracted, were compared using a three-way repeated-measures ANOVA with condition (motor/visual), subtask syllable (pa/ta/ka) and stimulus (pa/ta/ka) as within-subjects factors. The three-way interaction was significant (F(4,36) = 38.477, p < .001) (The main effects of condition and subtask syllable were significant (F(1,9) = 7.783, p < .05 and F(2,18) = 5.875, p < .05, respectively), while the main effect of stimulus was not significant (F(2,18) = 0.451, p > .05). All the two-way interactions (condition x subtask syllable, condition x stimulus, and subtask syllable x stimulus) were significant (F(2,18) = 13.985, p < .001, F(2,18) = 21.769, p < .001, and F(4,36) = 67.512, p < .001, respectively).) The simple main effect of condition (motor vs. visual) was significant for the six combinations of subtask syllable x stimulus where the subtask syllable was incongruent with the stimulus (Table 1). This effect was not significant for the three combinations where the subtask syllable was congruent with the stimulus. These results demonstrated that the perception of auditory stimuli was differently affected by the motor and visual subtasks if the subtask syllable was incongruent with the stimulus.

**Table 1:** Simple main effect of condition (motor vs. visual) for each combination of subtask syllable x stimulus.

auditory stim.	subtask syllable	F(1,9)	p
	pa	1.026	>.05
pa	ta	63.398	< .001
	ka	21.123	< .001
	pa	58.278	< .001
ta	ta	0.028	> .05
	ka	12.840	< .001
	pa	15.916	< .001
ka	ta	22.579	< .001
	ka	0.152	> .05

**Figure 3:**
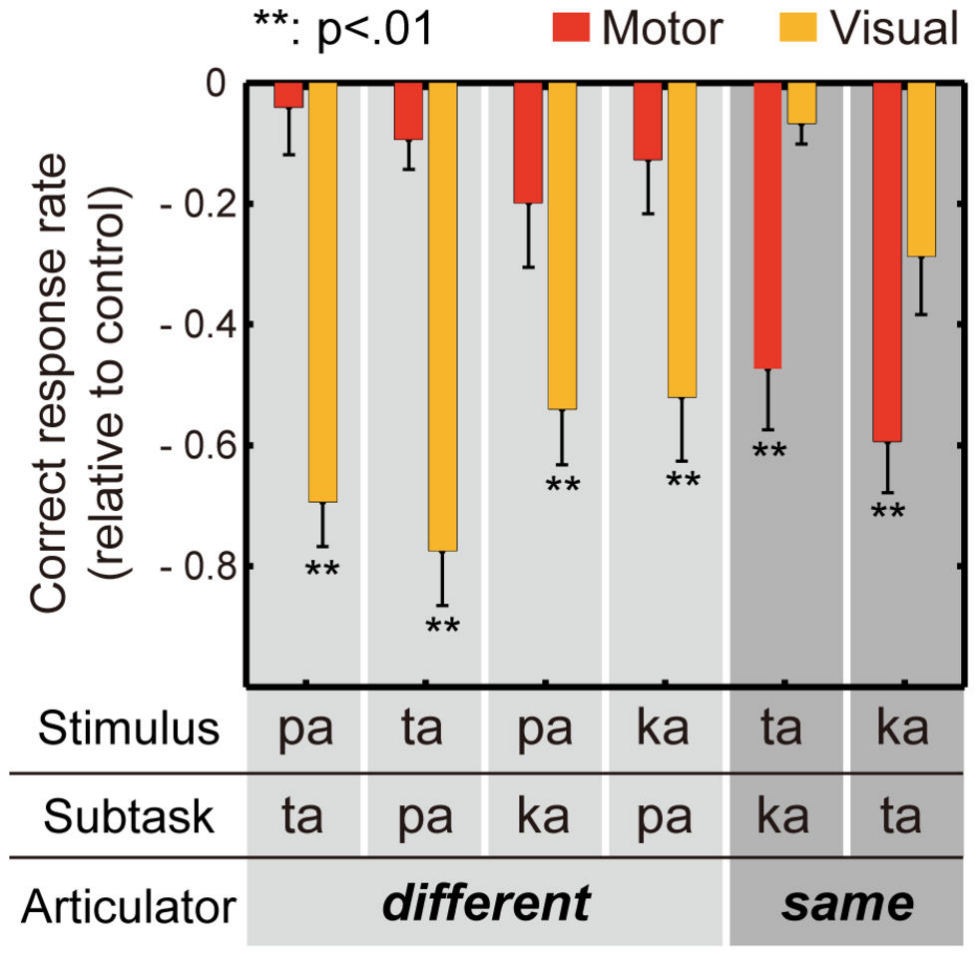
Effects of incongruent subtasks on syllable intelligibility. The mean and standard error (N = 10) of the correct response rates for auditory stimuli [pa], [ta], and [ka] when silently articulating (motor) and watching (visual) incongruent syllables, subtracted from their corresponding control levels, are shown.

The effect of the incongruent subtask syllable was further evaluated by comparing the correct response rates for each auditory stimulus under the control, motor, and visual conditions. A one-way repeated measures ANOVA was performed separately for each incongruent combination of auditory stimulus and subtask syllable, with condition (control/motor/visual) as a within-subjects factor (Table 2). The effect was significant for all combinations. Figure 3 shows the results of a post-hoc paired t-test with Bonferroni correction. A significant difference between motor and control conditions was found only for stimulus [ta] with motor [ka] and for stimulus [ka] with motor [ta] (p < .01). A significant difference between visual and control conditions was found for combinations other than [ta]-[ka] (p < .01). Given that the crucial articulator for the production of phoneme [p] is the lips whereas the tongue is crucial for [t] and [k], the results shown in Figure 3 can be interpreted as follows. The perception of the lip-related syllable ([pa]) was degraded by the visual tongue motions ([ta] and [ka]), and the perception of the tongue-related syllables ([ta] and [ka]) was degraded by the visual lip motion ([pa]). This pattern of audio-visual interaction replicates previous findings [23]. In contrast, the effect of a motor subtask revealed a distinct pattern of audio-articulatory interaction. The perception of the lip-related syllable ([pa]) was not affected by the tongue articulations ([ta] and [ka]), and the perception of the tongue-related syllables ([ta] and [ka]) was not affected by the lip articulation ([pa]). And more interestingly, the perception of the tongue-related syllables was degraded by the incongruent tongue articulation (audio [ta] by articulatory [ka], and vice versa). These results indicated that the contributions of the listener’s own articulatory movements and the other’s visual mouth movements to speech perception were different: the audio-visual interference occurred *across* different speech organs (between the lips and tongue), whereas the audio-articulatory interference occurred *within* the same organ (the tongue).

**Table 2:** Comparison of the correct response rates for auditory stimulus under control, motor and visual conditions for each incongruent combination of subtask syllable x stimulus.

auditory stim.	subtask syllable	F(2,18)	p
pa	ta	54.727	< .005
	ka	16.689	< .005
ta	pa	40.529	< .005
	ka	20.253	< .005
ka	pa	12.700	< .005
	ta	17.967	< .005

To verify whether the non-significant difference between the effects of visual [ta] and [ka] on perception was due to lack of their visual divergence, a visual-only control experiment was performed. Each of the three videos ([pa], [ta], and [ka]) used in the main experiments were presented 15 times, for a total of 45 trials, in a randomized order to another group of participants (N = 10, two female, mean age +/- SD = 33.8 +/- 8.3 years, all native speakers of Japanese). In each trial, the participants were asked to identify the syllable they observed in a 3-AFC ([pa], [ta], and [ka]) task. The correct response rates (mean +/- SE) were 0.98 +/- 0.014, 0.74 +/- 0.046, and 0.66 +/- 0.073 for [pa], [ta], and [ka], respectively. The results showed that there was a fairly clear difference between the visual stimuli [ta] and [ka].

One might expect that the effects of subtasks on the perception of voiced syllables (ba/da/ga) should be consistent with those of unvoiced syllables (pa/ta/ka). However, this was not the case because of a weak perception of [ga] under the control condition, due to the background noise with a 5 dB SNR: the correct response rates for [ba], [da], and [ga] were 0.82, 0.91, and 0.35, respectively. The lower baseline performance for the perception of [ga] compared with [ba] and [da] showed good agreement with the literature [25], thus reflecting the general properties of speech perception under constant noise. (Although an additional control experiment with a 10 dB SNR showed an accurate perception of [ga] (0.97), we regarded a 5 dB SNR as the most suitable noise level for our purpose because the performance for [pa], [ta], and [ka] at a 10 dB SNR reached its ceiling (0.97, 1.0, and 0.99, respectively), which prevented us from examining the possible “positive” effects of subtasks. Although the perception of [ta] still reached the ceiling even at a 5 dB SNR, a further increase in the noise level led to unacceptable deterioration in the baseline performance for all other syllables.)

We also analyzed the correct response rates for [ba], [da], and [ga] using the same procedure that we used for [pa], [ta], and [ka]. The correct response rates under the motor and visual conditions, from which the corresponding rates under the control condition were subtracted, were compared using a three-way repeated-measures ANOVA with condition (motor/visual), subtask syllable (pa/ta/ka) and stimulus (ba/da/ga) as within-subjects factors. The main effect of condition was not significant (F(1,9) = 0.105, p > .1), while the main effects of subtask syllable and stimulus were significant (F(2,18) = 5.472, p < .05 and F(2,18) = 13.405, p < .001, respectively). The two-way interaction of condition x subtask syllable was not significant (F(2,18) = 0.649, p > .1), while the two-way interactions of condition x stimulus and subtask syllable x stimulus were significant (F(2,18) = 12.816, p < .001 and F(4,36) = 5.618, p < .005, respectively). The three-way interaction was significant (F(4,36) = 22.482, p < .001). These results demonstrated that, with regard to hearing [ba], [da], and [ga], the difference between the effects of visual and motor subtasks was dependent on the stimuli type, unlike with [pa], [ta], and [ka]. When hearing voiced stimuli with silently articulating unvoiced syllables, the modes of the laryngeal and pharyngeal effectors associated with the stimuli were consistently incongruent with the actual states of those effectors of the listeners. In contrast, with regard to the visual subtask, the mouth video could provide only limited information about the modes of the laryngeal and pharyngeal effectors. This could be one reason why different results were obtained for unvoiced and voiced auditory stimuli.

## Discussion

Our experiment showed that the intelligibility of auditory syllables was degraded by *watching* syllable productions associated with a different primary articulator from that of the heard syllables, as expected (i.e., audio [p] by visual [t] and [k], and audio [t], [k] by visual [p]). In contrast, the syllable intelligibility was not degraded by *articulating* syllables associated with a different primary articulator, and instead was degraded only by *articulating* syllables that were incongruent but associated with the same primary articulator as that of the heard syllables (i.e., audio [t] by motor [k], and audio [k] by motor [t]). These results indicated that the perception of auditory syllables is influenced by the current state of a listener’s own speech organs, notably the lips and tongue. Our novel findings suggest that the perception of speech phonemes, which is associated with the activation of the articulator-specific motor brain areas, can be disturbed if those areas are simultaneously engaged in controlling the articulation of a different phoneme. We also showed that this audio-articulatory interaction is quite different from the well-known audio-visual interactions in speech perception, such as the McGurk effect. There has been a debate about whether such multisensory interactions in speech perception are mediated by an underlying motor representation of speech information. A study using functional imaging has shown that visual speech activates not only sensorimotor networks but also a much wider network possibly mediating a multisensory integration [26]. We found a clear dissociation between audio-articulatory and audio-visual interactions, implying that audio-visual speech perception is not a direct consequence of sensorimotor activity.

The three phonemes [p], [t], and [k] examined in the current study are all plosives (oral stop), where the airflow in the vocal tract is blocked and released by specific movements of the lips or tongue. Phonetically these phonemes are classified as labial (p), alveolar (t), and velar (k) plosives according to the place of articulation, i.e., where in the vocal tract (front, central, and back, respectively) the blockage is formed [24]. Despite their different places of articulation, our experimental results showed that labial articulation did not affect the perception of either the alveolar or the velar plosives, and neither alveolar nor velar articulation had any effect on the perception of the labial plosive. Of the three places of articulation, only labial articulation is associated with the movement of the lips, whereas the remaining two (alveolar and velar) are associated with tongue movements. And, in fact, the articulatory movement needed for pronouncing alveolar [t] disturbed the auditory perception of velar [k], and vice versa. The results suggest that the states of the tongue motor system affect the perception of tongue-related phonemes. Some studies using functional imaging and transcranial magnetic stimulation have found that speech perception modulates neural activity in motor speech areas in a somatotopic manner [13,14]. The articulator-dependent manner of the audio-articulatory interference effect observed in our study may be a reflection of such somatotopic linkage between the neural networks for speech production and perception.

In contrast, our experimental results indicated that the auditory degradation that occurred while watching speech depended largely on the visibility of the speech movements, rather than congruency between articulators. Thus, audio-visual speech integration is considered to occur with little access to the speech motor control. Several studies of brain activity during audio-visual speech perception have demonstrated the early visual modulation of the auditory cortex [27] and the left inferior frontal cortex [28]. Another report has revealed that the audio-visual speech illusion requires higher, conscious visual processing [29]. On the other hand, a transcranial magnetic stimulation study during audio-visual speech perception has shown that auditory and visual speech information can separately modulate excitability in the left tongue primary motor cortex in an early processing stage [30]. The audio-visual interactions influencing speech perception may therefore involve several distinct processing mechanisms, and should be further explored.

Although the involvement of the motor system in speech perception has been conceptually well described [31,32] and some studies have provided experimental evidence [33–37], there has been controversy regarding how incoming auditory information is processed by the motor nervous system and how it triggers a specific phoneme perception [38]. Although our experimental results suggest the existence of a direct link between the processes of speech motor control and phoneme perception, we have not examined at which stage of neural processing the audio-articulatory interaction occurs. It has been reported that the auditory cortical response to tones is suppressed both when seeing mouth articulation and when producing speech covertly, due to a top-down modulation from the motor speech system [39]. Thus, in the current study, articulatory imagery elicited during the preparation of silent articulation might have had a certain effect on the participants’ perceptual response. It will be important to determine whether articulatory imagery itself can have an organ-specific effect as observed in the current study. Furthermore, the motor control of speech articulation involves several stages such as motor planning, execution, or proprioceptive consequences. The syllable intelligibility changes observed in our study may reflect several different levels of integration between the neural representation of an auditory input and of articulatory movements. The organ-specific manner of the auditory-articulatory interference effect observed in our experiment is currently limited to the tongue-related syllables, because of the experimental requirements. We should further examine the effect on other speech effectors to make our findings more convincing.

## Supporting Information

File S1 The distributions of participants’ responses for every subtask are shown in Table S1-7 in the form of confusion matrices. In these tables auditory stimuli presented to the participants are listed vertically in the first column on the left. The participants’ responses are listed horizontally in the top row. The values in each cell indicate the mean (top) and variance (bottom) of percent responses (N = 10). The diagonal cells (highlighted in black) show the cases where the participants correctly perceived the auditory stimuli.
(DOC)
