# Supplementary material for: Speech Misperception: Speaking and Seeing Interfere Differently with Hearing
Source: PLoS One. 2013 Jul 3;8(7):e68619. doi: 10.1371/journal.pone.0068619 (PMC3701087; doi:10.1371/journal.pone.0068619)
Supplement: File S1 — The distributions of participants’ responses for every subtask are shown in Table S1-7 in the form of confusion matrices. In these tables auditory stimuli presented to the participants are listed vertically in the first column on the left. The participants’ responses are listed horizontally in the top row. The values in each cell indicate the mean (top) and variance (bottom) of percent responses (N = 10). The diagonal cells (highlighted in black) show the cases where the participants correctly perceived the auditory stimuli. (DOC) [file pone.0068619.s001.doc]

**Supporting Information**

**Table S1.** **Confusion matrix for control (auditory only) condition.**

|  |  | Response | | | | | | |
| --- | --- | --- | --- | --- | --- | --- | --- | --- |
|  |  | pa | ta | ka | ba | da | ga | a |
| Auditory stimulus | pa | 0.720 | 0.193 | 0.047 | 0.013 | 0.000 | 0.000 | 0.027 |
|  |  | 0.049 | 0.035 | 0.008 | 0.002 | 0.000 | 0.000 | 0.004 |
|  | ta | 0.000 | 0.993 | 0.007 | 0.000 | 0.000 | 0.000 | 0.000 |
|  |  | 0.000 | 0.000 | 0.000 | 0.000 | 0.000 | 0.000 | 0.000 |
|  | ka | 0.053 | 0.180 | 0.747 | 0.000 | 0.000 | 0.013 | 0.007 |
|  |  | 0.005 | 0.064 | 0.099 | 0.000 | 0.000 | 0.001 | 0.000 |
|  | ba | 0.180 | 0.000 | 0.000 | 0.820 | 0.000 | 0.000 | 0.000 |
|  |  | 0.039 | 0.000 | 0.000 | 0.039 | 0.000 | 0.000 | 0.000 |
|  | da | 0.000 | 0.060 | 0.013 | 0.000 | 0.907 | 0.020 | 0.000 |
|  |  | 0.000 | 0.011 | 0.002 | 0.000 | 0.021 | 0.001 | 0.000 |
|  | ga | 0.000 | 0.007 | 0.000 | 0.000 | 0.647 | 0.347 | 0.000 |
|  |  | 0.000 | 0.000 | 0.000 | 0.000 | 0.113 | 0.115 | 0.000 |
|  | a | 0.000 | 0.000 | 0.000 | 0.000 | 0.000 | 0.000 | 1.000 |
|  |  | 0.000 | 0.000 | 0.000 | 0.000 | 0.000 | 0.000 | 0.000 |

Table S2. Confusion matrix for motor condition with subtask syllable [pa].

|  |  | Response | | | | | | |
| --- | --- | --- | --- | --- | --- | --- | --- | --- |
|  |  | pa | ta | ka | ba | da | ga | a |
| Auditory stimulus | pa | 0.913 | 0.013 | 0.013 | 0.027 | 0.000 | 0.007 | 0.027 |
|  |  | 0.007 | 0.001 | 0.002 | 0.004 | 0.000 | 0.000 | 0.004 |
|  | ta | 0.087 | 0.900 | 0.013 | 0.000 | 0.000 | 0.000 | 0.000 |
|  |  | 0.026 | 0.025 | 0.001 | 0.000 | 0.000 | 0.000 | 0.000 |
|  | ka | 0.060 | 0.307 | 0.620 | 0.000 | 0.000 | 0.007 | 0.007 |
|  |  | 0.010 | 0.130 | 0.134 | 0.000 | 0.000 | 0.000 | 0.000 |
|  | ba | 0.380 | 0.007 | 0.007 | 0.600 | 0.007 | 0.000 | 0.000 |
|  |  | 0.062 | 0.000 | 0.000 | 0.055 | 0.000 | 0.000 | 0.000 |
|  | da | 0.000 | 0.047 | 0.000 | 0.013 | 0.940 | 0.000 | 0.000 |
|  |  | 0.000 | 0.011 | 0.000 | 0.001 | 0.010 | 0.000 | 0.000 |
|  | ga | 0.000 | 0.000 | 0.000 | 0.000 | 0.220 | 0.780 | 0.000 |
|  |  | 0.000 | 0.000 | 0.000 | 0.000 | 0.053 | 0.053 | 0.000 |
|  | a | 0.000 | 0.000 | 0.000 | 0.000 | 0.000 | 0.000 | 1.000 |
|  |  | 0.000 | 0.000 | 0.000 | 0.000 | 0.000 | 0.000 | 0.000 |

Table S3. Confusion matrix for motor condition with subtask syllable [ta].

|  |  | Response | | | | | | |
| --- | --- | --- | --- | --- | --- | --- | --- | --- |
|  |  | pa | ta | ka | ba | da | ga | a |
| Auditory stimulus | pa | 0.680 | 0.200 | 0.053 | 0.047 | 0.000 | 0.000 | 0.020 |
|  |  | 0.032 | 0.040 | 0.006 | 0.012 | 0.000 | 0.000 | 0.002 |
|  | ta | 0.014 | 0.979 | 0.007 | 0.000 | 0.000 | 0.000 | 0.000 |
|  |  | 0.002 | 0.002 | 0.000 | 0.000 | 0.000 | 0.000 | 0.000 |
|  | ka | 0.067 | 0.780 | 0.153 | 0.000 | 0.000 | 0.000 | 0.000 |
|  |  | 0.016 | 0.028 | 0.017 | 0.000 | 0.000 | 0.000 | 0.000 |
|  | ba | 0.173 | 0.007 | 0.000 | 0.813 | 0.000 | 0.007 | 0.000 |
|  |  | 0.025 | 0.000 | 0.000 | 0.021 | 0.000 | 0.000 | 0.000 |
|  | da | 0.000 | 0.200 | 0.000 | 0.013 | 0.787 | 0.000 | 0.000 |
|  |  | 0.000 | 0.046 | 0.000 | 0.001 | 0.045 | 0.000 | 0.000 |
|  | ga | 0.000 | 0.000 | 0.000 | 0.013 | 0.680 | 0.307 | 0.000 |
|  |  | 0.000 | 0.000 | 0.000 | 0.002 | 0.053 | 0.053 | 0.000 |
|  | a | 0.000 | 0.000 | 0.000 | 0.000 | 0.000 | 0.000 | 1.000 |
|  |  | 0.000 | 0.000 | 0.000 | 0.000 | 0.000 | 0.000 | 0.000 |

Table S4. Confusion matrix for motor condition with subtask syllable [ka].

|  |  | Response | | | | | | |
| --- | --- | --- | --- | --- | --- | --- | --- | --- |
|  |  | pa | ta | ka | ba | da | ga | a |
| Auditory stimulus | pa | 0.521 | 0.028 | 0.358 | 0.033 | 0.000 | 0.000 | 0.060 |
|  |  | 0.059 | 0.002 | 0.093 | 0.005 | 0.000 | 0.000 | 0.006 |
|  | ta | 0.033 | 0.520 | 0.440 | 0.000 | 0.000 | 0.000 | 0.007 |
|  |  | 0.007 | 0.109 | 0.123 | 0.000 | 0.000 | 0.000 | 0.000 |
|  | ka | 0.047 | 0.060 | 0.867 | 0.007 | 0.000 | 0.000 | 0.020 |
|  |  | 0.004 | 0.008 | 0.017 | 0.000 | 0.000 | 0.000 | 0.002 |
|  | ba | 0.213 | 0.000 | 0.000 | 0.780 | 0.000 | 0.007 | 0.000 |
|  |  | 0.032 | 0.000 | 0.000 | 0.031 | 0.000 | 0.000 | 0.000 |
|  | da | 0.000 | 0.067 | 0.000 | 0.000 | 0.893 | 0.040 | 0.000 |
|  |  | 0.000 | 0.036 | 0.000 | 0.000 | 0.033 | 0.003 | 0.000 |
|  | ga | 0.000 | 0.000 | 0.000 | 0.000 | 0.400 | 0.600 | 0.000 |
|  |  | 0.000 | 0.000 | 0.000 | 0.000 | 0.104 | 0.104 | 0.000 |
|  | a | 0.000 | 0.000 | 0.000 | 0.000 | 0.000 | 0.000 | 1.000 |
|  |  | 0.000 | 0.000 | 0.000 | 0.000 | 0.000 | 0.000 | 0.000 |

Table S5. Confusion matrix for visual condition with subtask syllable [pa].

|  |  | Response | | | | | | |
| --- | --- | --- | --- | --- | --- | --- | --- | --- |
|  |  | pa | ta | ka | ba | da | ga | a |
| Auditory stimulus | pa | 1.000 | 0.000 | 0.000 | 0.000 | 0.000 | 0.000 | 0.000 |
|  |  | 0.000 | 0.000 | 0.000 | 0.000 | 0.000 | 0.000 | 0.000 |
|  | ta | 0.761 | 0.219 | 0.013 | 0.007 | 0.000 | 0.000 | 0.000 |
|  |  | 0.080 | 0.084 | 0.002 | 0.000 | 0.000 | 0.000 | 0.000 |
|  | ka | 0.733 | 0.040 | 0.227 | 0.000 | 0.000 | 0.000 | 0.000 |
|  |  | 0.099 | 0.008 | 0.107 | 0.000 | 0.000 | 0.000 | 0.000 |
|  | ba | 0.147 | 0.000 | 0.000 | 0.853 | 0.000 | 0.000 | 0.000 |
|  |  | 0.020 | 0.000 | 0.000 | 0.020 | 0.000 | 0.000 | 0.000 |
|  | da | 0.007 | 0.000 | 0.000 | 0.300 | 0.680 | 0.013 | 0.000 |
|  |  | 0.000 | 0.000 | 0.000 | 0.154 | 0.159 | 0.001 | 0.000 |
|  | ga | 0.000 | 0.000 | 0.000 | 0.140 | 0.080 | 0.779 | 0.000 |
|  |  | 0.000 | 0.000 | 0.000 | 0.085 | 0.043 | 0.158 | 0.000 |
|  | a | 0.073 | 0.000 | 0.000 | 0.140 | 0.000 | 0.000 | 0.787 |
|  |  | 0.030 | 0.000 | 0.000 | 0.076 | 0.000 | 0.000 | 0.174 |

Table S6. Confusion matrix for visual condition with subtask syllable [ta].

|  |  | Response | | | | | | |
| --- | --- | --- | --- | --- | --- | --- | --- | --- |
|  |  | pa | ta | ka | ba | da | ga | a |
| Auditory stimulus | pa | 0.027 | 0.940 | 0.013 | 0.007 | 0.007 | 0.000 | 0.007 |
|  |  | 0.007 | 0.011 | 0.002 | 0.000 | 0.000 | 0.000 | 0.000 |
|  | ta | 0.000 | 0.993 | 0.007 | 0.000 | 0.000 | 0.000 | 0.000 |
|  |  | 0.000 | 0.000 | 0.000 | 0.000 | 0.000 | 0.000 | 0.000 |
|  | ka | 0.000 | 0.540 | 0.460 | 0.000 | 0.000 | 0.000 | 0.000 |
|  |  | 0.000 | 0.173 | 0.173 | 0.000 | 0.000 | 0.000 | 0.000 |
|  | ba | 0.020 | 0.240 | 0.000 | 0.267 | 0.440 | 0.027 | 0.007 |
|  |  | 0.001 | 0.056 | 0.000 | 0.105 | 0.061 | 0.003 | 0.000 |
|  | da | 0.000 | 0.007 | 0.000 | 0.000 | 0.987 | 0.007 | 0.000 |
|  |  | 0.000 | 0.000 | 0.000 | 0.000 | 0.001 | 0.000 | 0.000 |
|  | ga | 0.000 | 0.000 | 0.000 | 0.000 | 0.187 | 0.813 | 0.000 |
|  |  | 0.000 | 0.000 | 0.000 | 0.000 | 0.110 | 0.110 | 0.000 |
|  | a | 0.000 | 0.000 | 0.000 | 0.000 | 0.000 | 0.000 | 1.000 |
|  |  | 0.000 | 0.000 | 0.000 | 0.000 | 0.000 | 0.000 | 0.000 |

**Table S7. Confusion matrix for visual condition with subtask syllable [ka].**

|  |  | Response | | | | | | |
| --- | --- | --- | --- | --- | --- | --- | --- | --- |
|  |  | pa | ta | ka | ba | da | ga | a |
| Auditory stimulus | pa | 0.180 | 0.467 | 0.307 | 0.007 | 0.007 | 0.000 | 0.033 |
|  |  | 0.055 | 0.060 | 0.061 | 0.000 | 0.000 | 0.000 | 0.004 |
|  | ta | 0.000 | 0.927 | 0.073 | 0.000 | 0.000 | 0.000 | 0.000 |
|  |  | 0.000 | 0.016 | 0.016 | 0.000 | 0.000 | 0.000 | 0.000 |
|  | ka | 0.000 | 0.060 | 0.900 | 0.000 | 0.000 | 0.013 | 0.027 |
|  |  | 0.000 | 0.003 | 0.014 | 0.000 | 0.000 | 0.002 | 0.004 |
|  | ba | 0.033 | 0.187 | 0.027 | 0.340 | 0.327 | 0.087 | 0.000 |
|  |  | 0.002 | 0.044 | 0.004 | 0.107 | 0.053 | 0.019 | 0.000 |
|  | da | 0.000 | 0.013 | 0.000 | 0.000 | 0.980 | 0.007 | 0.000 |
|  |  | 0.000 | 0.002 | 0.000 | 0.000 | 0.002 | 0.000 | 0.000 |
|  | ga | 0.000 | 0.000 | 0.000 | 0.000 | 0.100 | 0.900 | 0.000 |
|  |  | 0.000 | 0.000 | 0.000 | 0.000 | 0.086 | 0.086 | 0.000 |
|  | a | 0.000 | 0.000 | 0.000 | 0.000 | 0.000 | 0.000 | 1.000 |
|  |  | 0.000 | 0.000 | 0.000 | 0.000 | 0.000 | 0.000 | 0.000 |
